# A rapid and simple non-radioactive assay for measuring uptake by solute carrier transporters

**DOI:** 10.3389/fphar.2024.1355507

**Published:** 2024-04-24

**Authors:** Kunling Song, Longbin Zhang, Xian Fu, Linfeng Li, Gaolin Zhu, Mingjun Wu, Wei Zhang, Jia He, Sanyong Zhu, Yongjun Dang, Jun-Yan Liu, Chang Chen, Zufeng Guo

**Affiliations:** ^1^ Basic Medicine Research and Innovation Center for Novel Target and Therapeutic Intervention (Ministry of Education), Institute of Life Sciences and Department of Breast and Thyroid Surgery, the Second Affiliated Hospital, Chongqing Medical University, Chongqing, China; ^2^ Basic Medicine Research and Innovation Center for Novel Target and Therapeutic Intervention (Ministry of Education), Institute of Life Sciences and Department of Anesthesiology, the Second Affiliated Hospital, Chongqing Medical University, Chongqing, China; ^3^ Institute of Life Sciences, Chongqing Medical University, Chongqing, China; ^4^ College of Pharmacy, Chongqing Medical University, Chongqing, China

**Keywords:** solute carrier transporter, uptake assay, LC-MS/MS, LAT1, NTCP

## Abstract

**Introduction:** Solute carrier (SLC) transport proteins play a crucial role in maintaining cellular nutrient and metabolite homeostasis and are implicated in various human diseases, making them potential targets for therapeutic interventions. However, the study of SLCs has been limited due to the lack of suitable tools, particularly cell-based substrate uptake assays, necessary for understanding their biological functions and for drug discovery purposes.

**Methods:** In this study, a cell-based uptake assay was developed using a stable isotope-labeled compound as the substrate for SLCs, with detection facilitated by liquid chromatography-tandem mass spectrometry (LC-MS/MS). This assay aimed to address the limitations of existing assays, such as reliance on hazardous radiolabeled substrates and limited availability of fluorescent biosensors.

**Results:** The developed assay was successfully applied to detect substrate uptakes by two specific SLCs: L-type amino acid transporter 1 (LAT1) and sodium taurocholate co-transporting polypeptide (NTCP). Importantly, the assay demonstrated comparable results to the radioactive method, indicating its reliability and accuracy. Furthermore, the assay was utilized to screen for novel inhibitors of NTCP, leading to the identification of a potential NTCP inhibitor compound.

**Discussion:** The findings highlight the utility of the developed cell-based uptake assay as a rapid, simple, and environmentally friendly tool for investigating SLCs’ biological roles and for drug discovery purposes. This assay offers a safer alternative to traditional methods and has the potential to contribute significantly to advancing our understanding of SLC function and identifying therapeutic agents targeting SLC-mediated pathways.

## 1 Introduction

Transport of solutes by protein transporters is essential for many biological processes, such as nutrient uptake, ion transport, and waste removal. With more than 450 members, solute carriers (SLCs) represent the biggest group of transporters and the second largest family of membrane proteins after G-protein-coupled receptors (GPCRs) in the human genome (Hoglund et al., 2011). SLCs mediate the movement of various substances, including amino acids, sugars, nucleotides, and also small molecule drugs, across both cellular and organellar membranes (Zhang et al., 2019; Girardi et al., 2020). At least half of the SLCs are associated with human diseases, including cancer, diabetes, and central nervous system disorders. For example, cancer cells have a high demand for glucose and amino acids as nutrients due to rapid cell growth and as a consequence, upregulate a subset of isoforms of glucose transporters (GLUTs) and L-type amino acid transporters (LATs) (Hay, 2016; Papalazarou and Maddocks, 2021). GLUT1 (encoded by SLC2A1) and LAT1 (encoded by SLC7A5) have served as promising targets for discovering anticancer agents in recent years (Scalise et al., 2018; Holman, 2020). In addition, mutations in LAT1 lead to neurological and behavioral abnormalities associated with autism spectrum disorder (Tarlungeanu et al., 2016). Moreover, several SLCs are acting as cellular receptors for the entry of viruses. For example, sodium taurocholate co-transporting polypeptide (NTCP, encoded by SLC10A1) is a liver bile acids transporter and has also been shown to be a functional receptor for both hepatitis B virus (HBV) and hepatitis D virus (HDV) (Yan et al., 2012).

In recent years, there is increasing interest in elucidating physiological roles and therapeutic potential of SLCs (Lin et al., 2015; Wang et al., 2020). However, compared to GPCRs, the majority of SLCs remains understudied, partially due to lack of suitable tools, especially cell-based substrate uptake assays (Cesar-Razquin et al., 2015). At present, four types of cell-based substrate uptake assays have been reported to assess SLC transport function, including radioligand uptake assay, fluorescent substrate uptake assay, genetically encoded fluorescent biosensor assay, and mass spectrometry-based transport assays (Dvorak et al., 2021). Radioligand uptake assays employ radiolabeled substrates, typically ^3^H or ^14^C labeled, to quantitatively study the substrate uptake across the plasma membrane (Sucic et al., 2016). However, radiolabeled substrates are hazardous, costly and require special handling and authorizations, which limit the usage of these assays. Fluorescent substrate uptake assays usually rely on transport of fluorescently labeled substrates of SLCs (Fardel et al., 2015). Although this strategy circumvents the use of radiolabeled substrates, it requires the identification of a suitable fluorescent surrogate substrate, which is not feasible for every SLC. Genetically encoded fluorescent biosensor assays express proteins that sense a physical property or bind an analyte, and then translate its concentration into a change in fluorescence (Keller et al., 2021). The major limitation of this strategy is the availability of biosensors because most of biosensors have been developed to investigate signaling events but not for transport measurements. Mass spectrometry-based transport assays were widely used in metabolomics analysis (Wright Muelas et al., 2020). However, both targeted and untargeted metabolomics require expensive instrumentation with specialist knowledge, along with complex data processing and analysis. Therefore, there is a critical need for the development of a rapid, simple, and environmental-friendly cell-based transport assay to elucidate functional roles and therapeutic potential of SLCs.

In the current study, we employed a stable isotope labeled compound as SLC substrate and LC-MS/MS as the detection method to develop a novel cell-based uptake assay for SLCs. We successfully applied this assay to detect leucine-d_3_ uptake by LAT1 and taurocholic acid (TCA)-d_4_ uptake by NTCP. In comparison to the radioactive method, our stable isotope assay yields similar IC_50_ values for LAT1 inhibitor JPH203 and NTCP inhibitor myrcludex B. Moreover, we employed this assay to screen numbers of rapamycin-inspired hybrid macrocycles called rapafucins for inhibitors of NTCP and found one compound as a potential novel NTCP inhibitor. Together, our assay could be an alternative to the currently available cell-based uptake assays for SLCs.

## 2 Materials and methods

### 2.1 Chemicals and reagents

Taurocholic acid-d_4_ (TCA-d_4_) and L-leucine-5,5,5-d_3_ (leucine-d_3_) were purchased from Cayman (MI, United States). Taurocholic acid-[^3^H(G)] (^3^H-TCA) and L-[3,4,5-^3^H(N)]-leucine (^3^H-leucine) were purchased from PerkinElmer (MA, United States). JPH203 was purchased from Selleck (Shanghai, China). Myrcludex B was a gift from Dr. Ailong Huang’s Laboratory. CellTiter-Glo^®^ and Bright-Glo™ reagents were purchased from Promega (WI, United States). Membrane protein extraction kit (no. 89842) was purchased from ThermoFisher Scientific (MA, United States). LAT 1 antibody (no.5347, 1:1,000) and His-Tag antibody (no.12698, 1:3,000) were purchased from Cell Signaling Technology (MA, United States). EGFR antibody (no. 66455-1-1G, 1:1,000) and HSP90 antibody (no. 13171-1-AP, 1:1,000) were purchased from Proteintech (IL, United States). Mouse anti-rabbit IgG-HRP (no.2357, 1:5,000) was purchased from Santa Cruz Biotechnology (TX, United States).

### 2.2 Cell culture

All cells were grown at 37 C with 5% CO_2_ in a humidified environment. LS174T and LS174T LAT1 knockout cells were grown in EMEM media with 10% FBS. HepG2 wildtype and HepG2 NTCP cells were grown in DMEM media with the addition of 10% FBS.

### 2.3 Membrane protein extraction and western blot analysis

Membrane proteins were isolated from LS174T and HepG2 NTCP cells following the membrane protein extraction kit protocol. Membrane and cytosolic fractions were separated by SDS-PAGE and transferred to a nitrocellulose membrane. Membranes were first blocked in 5% (wt/vol) BSA in Tris-buffered saline plus 0.1% Tween 20 (TBST) at room temperature for 30 min and incubated with primary antibodies at 4 °C for overnight. Membranes were then washed three times with TBST and incubated with secondary antibodies at room temperature for another 1 h. Membranes were washed with TBST three times again and incubated with ECL substrate for 1.5 min at room temperature. Pictures were captured using a CLINX Image Station.

### 2.4 Preparation of SLC7A5 gene knock-out cell line

CRISPR-Cas9 technology was applied to create SLC7A5 gene knock-out cell line. Briefly, guide RNAs targeting SLC7A5 (Table 1) were individually cloned into Lentiviral V2 vector. The gene product was transfected into HEK293T cells with pSPAX2 and pMD2G using lipofectamine 2,000 and lentiviruses were harvested after 72 h LS174T cells were infected with the corresponding lentivirus for 48 h. Cells were then selected with 10 μg/mL of puromycin for 2 weeks and maintained at the same concentration of antibiotic for culture. Efficiency of SLC7A5 gene knock out was evaluated by western blot after 10 days of transduction and puromycin selection using anti-LAT1 antibody.

**TABLE 1:** gRNA sequences for LAT1 (SLC7A5) gene knockout.

Gene	gRNA
SLC7A5	CGA​CTA​CGC​CTA​CAT​GCT​GG
SLC7A5	CGG​AAC​ATC​ACG​CTG​CTC​AA
SLC7A5	CGT​GAA​CTG​CTA​CAG​CGT​GA
SLC7A5	ATT​GTG​CTG​GCA​TTA​TAC​AG
SLC7A5	CTT​CTT​CAA​CTG​GCT​CTG​CG

### 2.5 L-leucine-5,5,5-d_3_ (leucine-d_3_) uptake assay

LS174T cells were seeded into 12-well plates at a density of 1×10^6^/well. After a 48 h incubation, cells were carefully washed with prewarmed Na^+^-free uptake buffer (10 mM Tris-HCl, 150 mM KCl, 5 mM MgCl_2_, 1 mM EGTA, pH 7.4) twice and incubated in 0.5 mL of Na^+^-free uptake buffer at 37^o^C for 15 min. Cells were washed with Na^+^-free uptake buffer once again and incubated with different concentrations of JPH203 or DMSO at 37^o^C for 15 min. Cells were then allowed to take up 5 μM of leucine-d_3_ at 37^o^C for another 15 min. After incubation, cells were washed with Na^+^-free uptake buffer three times and lysed with 200 μL of acetonitrile. The cell lysate was transferred to a new tube and the supernatant was collected by centrifugation at 12,000 rpm for 10 min at 4°C. Leucine-d_3_ uptake was quantified with LC-MS/MS.

### 2.6 Taurocholic acid-d_4_ (TCA-d_4_) uptake assay

HepG2 NTCP cells were seeded into 12-well plates at a density of 2×10^5^/well. After a 48 h incubation, cells were carefully washed with uptake buffer (10 mM HEPES, 100 mM NaCl, 2 mM KCl, 1 mM MgCl_2_, 1 mM CaCl_2_, pH 7.4) twice and incubated in 0.5 mL of uptake buffer for 15 min at 37^o^C. Cells were washed with uptake buffer once again and incubated with different concentrations of myrcludex B or DMSO at 37^o^C for 15 min. Cells were then allowed to take up 5 μM of TCA-d_4_ at 37^o^C for another 15 min. After incubation, cells were washed with uptake buffer three times and lysed with 200 μL of ethanol. The cell lysate was transferred to a new tube and the supernatant was collected by centrifugation at 12,000 rpm for 10 min at 4°C. TCA-d_4_ uptake was quantified with LC-MS/MS.

### 2.7 LC-MS/MS conditions

Prepared samples were separated on an Agilent 1290 II system (CA, United States) equipped with an XSelect HSS T3 C_18_ reverse phase column (2.1 × 150 mm, 3.5 μm). To separate leucine-d_3_, the mobile phase consisted of solution A (0.1% formic acid in water) and solution B (0.1% formic acid in acetonitrile). To separate TCA-d_4_, the mobile phase consisted of solution A (5 mM ammonium formate) and solution B (methanol). Separations were achieved using the following conditions: 0–3 min 70%–95% B; 3–5 min 95% B; 5–5.1 min 95%–70% B; 5.one to six min 70% B. The flow rate was 0.4 mL/min and the injection volume of each sample was 5 uL.

Targeted analytes were monitored in a negative multiple-reaction monitoring (MRM) mode on an Agilent 6495C triple quadrupole LS/MS system (CA, United States). The ion spray voltage was set as −4500 V and the source temperature 450°C. The gas flow was fixed for curtain gas, ion source gas 1, and ion source gas 2 as 40, 50, and 50 L/h, respectively, and the collision gas was set as medium. Leucine-d_3_ and TCA-d_4_ were monitored in negative ion mode with MRM ion pairs at 135.1 > 89.2 (collision energy: 14 eV) and 518.0 > 124.0 (collision energy: 65 eV), respectively. Both calibrators used for Leucine-d_3_ and TCA-d_4_ were 41 nM, 123 nM, 370 nM, 1111 nM, 3333 nM, and 10,000 nM. Peak areas were used to reflect concentrations in the supernatants. Analyst 1.7.2 software was used for system operation, data acquisition, and data processing.

### 2.8 L-[3,4,5-^3^H(N)]-leucine (^3^H-leucine) uptake assay

The inhibitory activity of JPH203 on LAT1 was analyzed by measuring the cell uptake of ^3^H-leucine as previously described with minor modifications (Oda et al., 2010). Similar to leucine-d_3_ uptake assay, LS174T cells were washed twice and incubated in Na^+^-free uptake medium (10 mM Tris-HCl, 150 mM KCl, 5 mM MgCl_2_, 1 mM EGTA, pH 7.4) for 15 min. Cells were added with JPH203 or DMSO and incubated for another 15 min before adding ^3^H-leucine. After 15 min incubation, cells were washed three times and lysed with 0.2 N NaOH plus 0.2% SDS. The cell lysate was transferred to a new tube containing 1 mL of optiphase supermix. ^3^H-leucine uptake was quantified with a scintillation counter.

### 2.9 Taurocholic acid-[^3^H(G)] (^3^H-TCA) uptake assay

The inhibitory activity of myrcludex B (Gripon et al., 2005) on NTCP was analyzed by measuring the cell uptake of ^3^H-TCA as previously described with minor modification (Asami et al., 2022). Similar to TCA-d4 uptake assay, HepG2 NTCP cells were washed twice and incubated in uptake medium (10 mM HEPES, 100 mM NaCl, 2 mM KCl, 1 mM MgCl_2_, 1 mM CaCl_2_, pH 7.4) for 15 min. Cells were added with myrcludex B or DMSO and incubated for another 15 min before adding ^3^H-TCA. After 15 min of incubation, the cells were washed for three times and lysed with 0.2 N NaOH plus 0.2% SDS. The cell lysate was transferred to a new tube containing 1 mL of optiphase supermix. ^3^H-Leucine uptake was quantified with a scintillation counter.

### 2.10 Cell viability assay

HepG2 NTCP cells were seeded at a density of 1,000 cells/well in 96-well plates. After an overnight recovery, compounds were added. Following a 72 h incubation, 100 μL of the culture medium was removed, and each well was added 40 μL of CellTiter-Glo^®^ reagent. The 96 well-plates were then placed on an oscillator with gentle shaking to mix for 10 min at room temperature. The chemiluminescent signals were detected using a multimode plate reader (EnSight™, PerkinElmer, United States).

### 2.11 Molecular modeling of NTCP-JH7 interaction

JH7 was ionized at pH 7.0 ± 0.5 using Epik module to generate reasonable states. Macrocycle conformational sampling was then implemented with Prime module. The sampling intensity was set to 10,000 to generate conformers with a high diversity of rings. The top 20% of conformers were energetically optimized to enter molecular docking stage. The binding pocket prediction was carried out in SiteMap module using a recently reported cryo-EM structure of NTCP-preS1 complex (PDB code: 8HRX) (Asami et al., 2024). Considering the relatively large size of ligand, detection of shallow binding sites option was enabled to identify top-ranked pockets. The prepared set of JH7 conformers was docked to the predicted pocket of NTCP using Glide module. Receptor grid box was generated and then applied to the first round of docking using SP protocol with the sample ring conformations option disabled. Top 10% of structures were further simulated using XP protocol to achieve high-accuracy prediction. The final poses were ranked and visually assessed to provide the most probable binding mode.

### 2.12 BMP reporter gene assay

HEK293T cells transfected with Id1-Luc reporter construct were seeded into a 96-well plate (190 μL/well of 3,000 cells/mL)) and then treated with 3 μM JH7 for 12 h. FK506 and DMSO were used as positive and negative controls, respectively. Cells were removed 100 μL DMEM and added in 40 μL Bright-Glo™ Luciferase reagent (per well) and placed on a plate-shaker for 10 min. Bright-Glo™ Luciferase Assay was performed with Ensight plate reader.

## 3 Results and discussion

Radioligand uptake assay is the most commonly used strategy to assess SLC transport function and has been employed to study diverse SLC families. However, a disadvantage of this assay is that radioactive materials are hazardous, costly and require special handling and authorizations. In comparison to radiolabeled substrates, stable isotope labeled substrates offer several advantages including non-radioactivity, less costly, non-toxicity and easy accessibility. In addition, stable isotope labeled substrates usually serves as internal standards in LC-MS/MS bioanalysis, suggesting that they could be easily separated and analyzed by LC-MS/MS. The existence of these advantages raised the intriguing question of whether using stable isotope labeled substrate as SLC substrate and LC-MS/MS as the detection method could develop an alternative to radioligand uptake assay for SLC. Although several uptake assays using stable isotope labeled substrates have been applied to study SLC functions, none has reported both the optimization of assay and the comparison to the radioactive method (Gauthier-Coles et al., 2021; Bay et al., 2022; Papalazarou et al., 2023).

**FIGURE 1:**
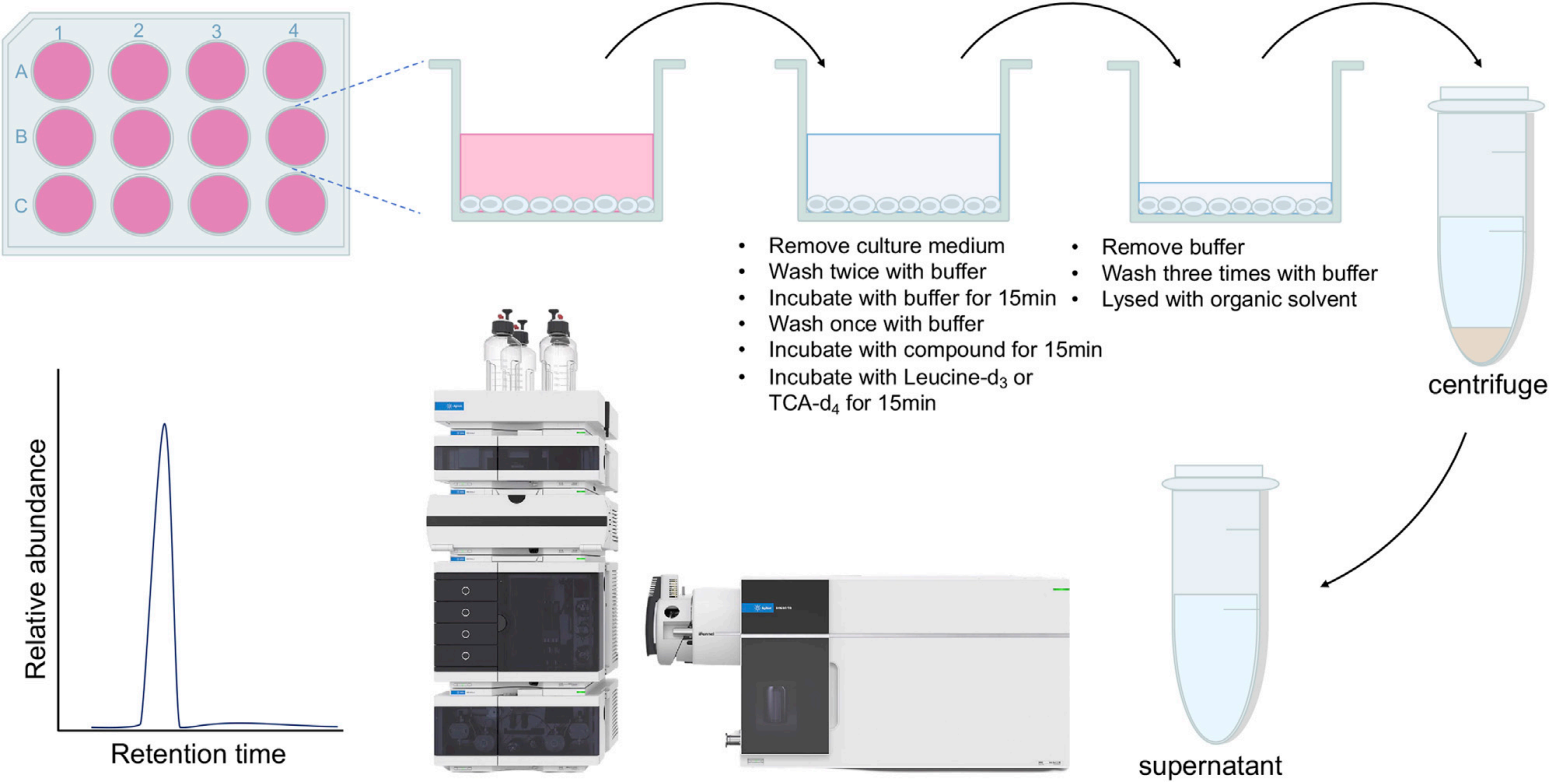
Flow chart of the uptake assay proposed in this study.

**FIGURE 2:**
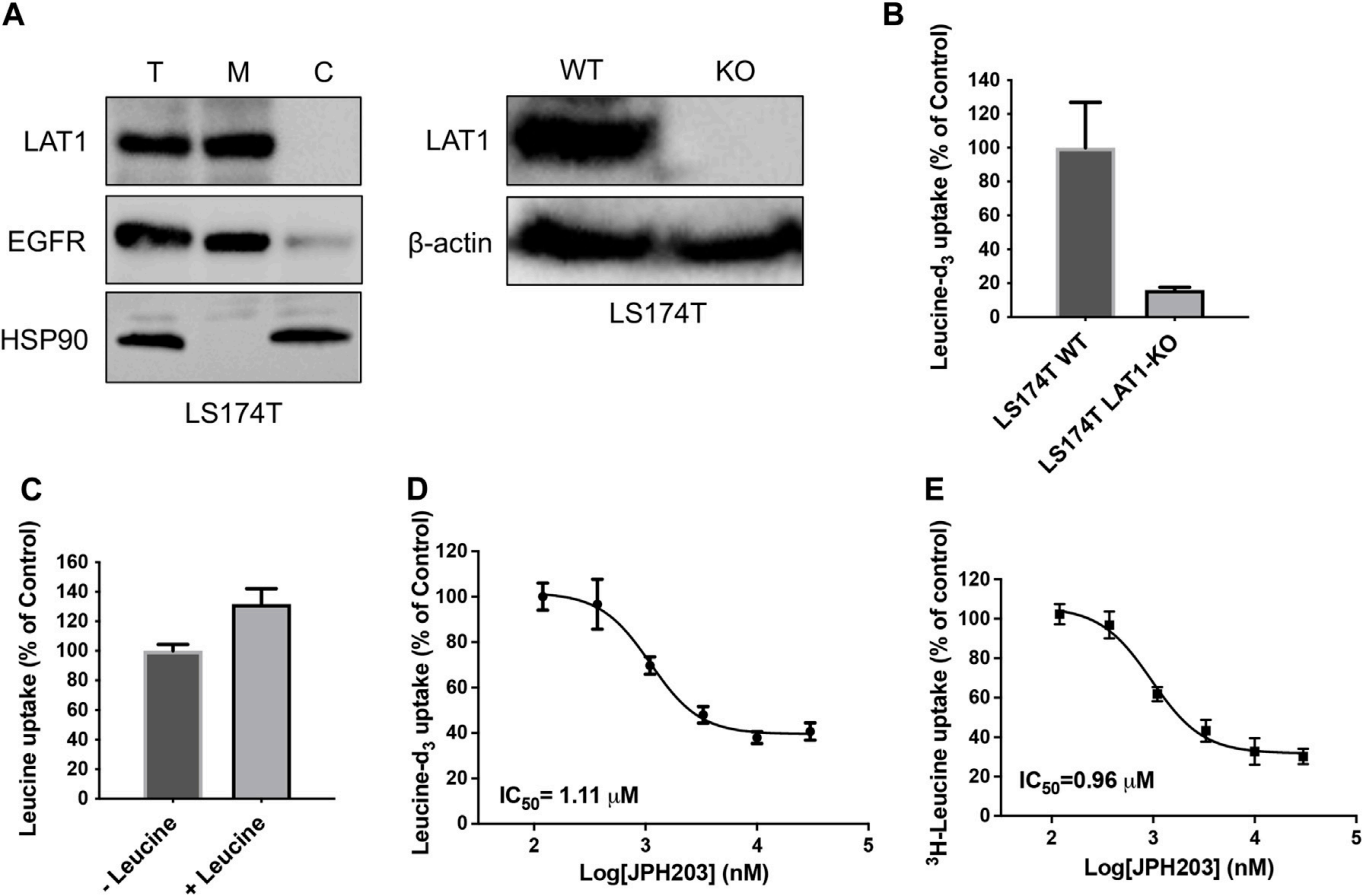
Development of leucine-d_3_ uptake assay for LAT1. **(A)** Western blot results show LAT1 is expressed on the cell surface (left) and LAT1 gene is successfully knocked out in LS174T cells (right). T stands for total protein; M stands for membrane fraction; C stands for cytosolic fraction; WT stands for wild type; KO stands for knock out. EGFR serves as a loading control for membrane proteins; HSP90 serves as a loading control for cytosolic proteins. β-actin serves as a loading control for total proteins. **(B)** Wild type LS174T cells show strong leucine-d_3_ uptake, while LS174T LAT1 gene knockout cells lost uptake activity. **(C)** Adding 5 μM of leucine as uptake substrate did not significantly increase the signal of mass spectrometry. **(D)** Dose dependent inhibition of LAT1 transport activity by JPH203 determined by leucine-d_3_ uptake assay. **(E)** Dose dependent inhibition of LAT1 transport activity by JPH203 determined by ^3^H-leucine uptake assay. Error bars represent s.d. Data are presented as mean ± s.d; *n* = 3 independent experiments.

In a continuing effort to explore the possibility to use stable isotope labeled substrate to develop SLC uptake assay, we first attempted to develop leucine-d_3_ uptake assay for LAT1. LAT1 belongs to the SLC superfamily and enables the transport of large neutral essential amino acids including leucine into cells. Dysfunctions of LAT1 have been reported to be correlated with several human diseases including cancer and autism spectrum disorder (Tarlungeanu et al., 2016; Scalise et al., 2018). Extensive efforts have been made to discover new inhibitors of LAT1 as leads for developing novel anti-cancer drugs. Development of novel cell-based uptake assay could facilitate the functional study of LAT1 and the characterization of novel LAT1 inhibitors. To develop leucine-d_3_ uptake assay, we first optimized the collision energy to achieve the optimal MRM ion pair for monitoring leucine-d_3_ and then adjusted mobile phases and gradient condition for LC to achieve a satisfactory separation. LAT1 has been shown to be the only functional large neutral amino acid transporter expressed in human colon adenocarcinoma LS174T cell line (Cormerais et al., 2016). Using LS174T cells, we next optimized uptake assay conditions by adjusting uptake buffer, incubation time and extraction solvent. The protocol for the uptake assay proposed in this study is illustrated in Figure 1. To demonstrate the uptake specificity of LAT1 in LS174T cells, we also knocked out SLC7A5 gene using CRISPR-Cas9 technology. Western blot analysis confirmed that LAT1 is expressed on the cell surface and SLC7A5 gene was successfully knocked out in LS174T cells (Figure 2A). Using optimized LC-MS/MS and uptake conditions, we performed both leucine-d_3_ and leucine uptake assays on LS174T wildtype and SLC7A5 gene knocked out cells. As expected, LS174T wildtype cells were able to uptake leucine-d_3_, while deletion of LAT1 abolished the uptake activity (Figure 2B). Unlike leucine-d_3_, adding unlabeled leucine only increased about 30% of signal in mass spectrometry due to the high concentration of endogenous leucine in LS174T cells, suggesting that leucine-d_3_ is a better substrate than unlabeled leucine to develop uptake assay for LAT1 (Figure 2C). To test whether this assay could be used to characterize modulators of SLCs, we determine the inhibitory activity of LAT1 inhibitor JPH203 (Oda et al., 2010) in LS174T cells. We observed that JPH203 dose-dependently inhibited leucine-d_3_ uptake in LS174T cells with an IC_50_ value of 1.11 ± 0.21 μM (Figure 2D). As a comparison, we also measured the impact of JPH203 in LS174T cells using previously reported ^3^H-leucine uptake assay (Figure 2E). Importantly, ^3^H-leucine uptake assay yielded the similar result with IC_50_ value of 0.96 ± 0.09 μM, thus indicating that our assay was capable of measuring uptake by LAT1 and could be an alternative to ^3^H-leucine uptake assay.

**FIGURE 3:**
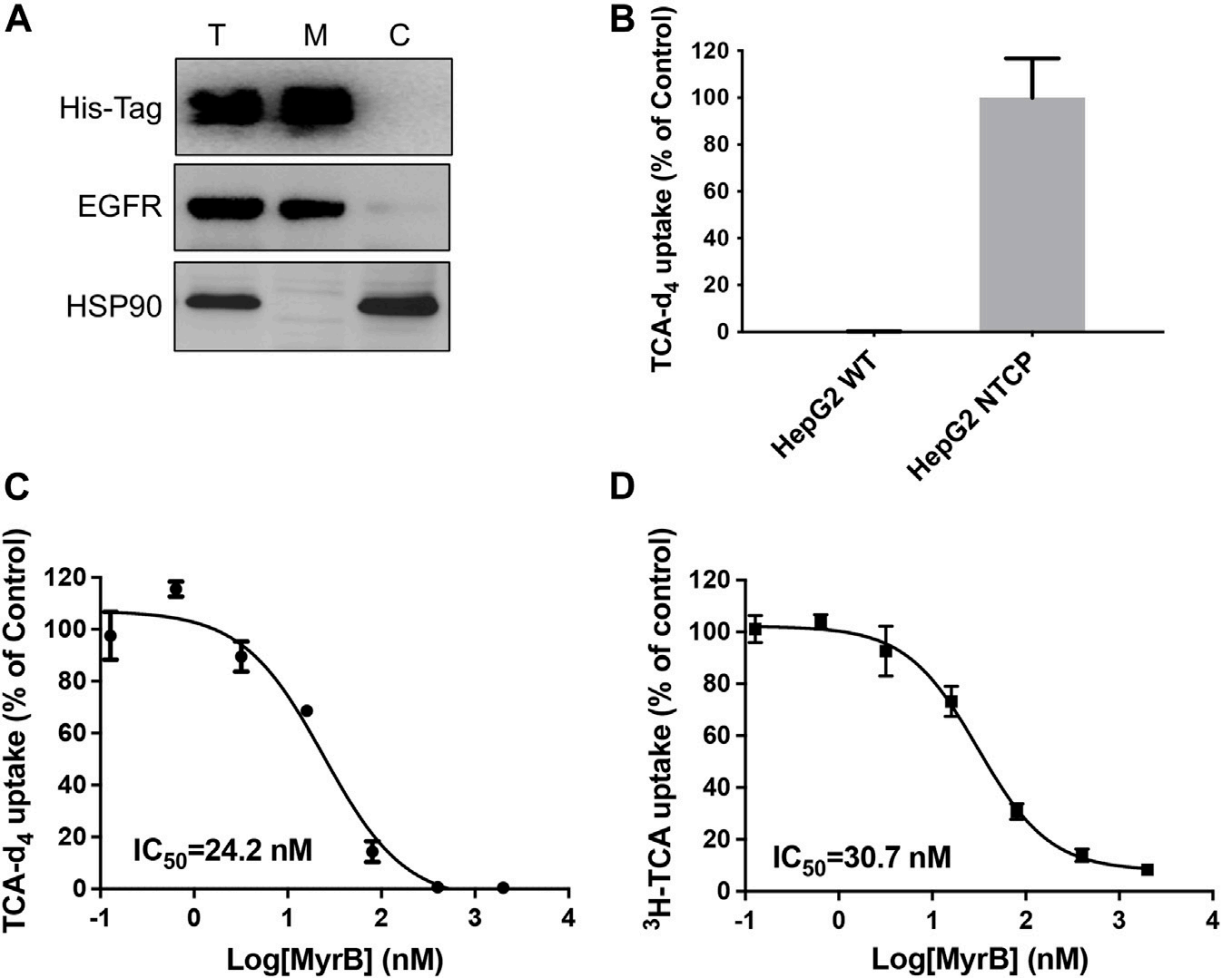
Development of TCA-d_4_ uptake assay for NTCP. **(A)** Western blot result shows His-tagged NTCP is expressed on the cell surface in HepG2 NTCP cells. T stands for total protein; M stands for membrane fraction; C stands for cytosolic fraction. EGFR serves as a loading control for membrane proteins; HSP90 serves as a loading control for cytosolic proteins. **(B)** HepG2 NTCP cells show strong TCA-d_4_ uptake, while HepG2 wildtype cells do not. **(C)** Dose dependent inhibition of NTCP transport activity by myrcludex B determined by TCA-d_4_ uptake assay. **(D)** Dose dependent inhibition of NTCP transport activity by myrcludex B determined by ^3^H-TCA uptake assay. Error bars represent s.d. Data are presented as mean ± s.d; *n* = 3 independent experiments.

To determine whether our assay could be used to other SLCs, we next develop TCA-d_4_ uptake assay for NTCP. Like LAT1, NTCP also belongs to SLC superfamily and is expressed on the hepatic basolateral membranes, functioning for bile acids uptake into hepatocytes. NTCP plays an essential role in both HBV and HDV entry into host cells and has been pursued as promising anti-HBV/HDV drug targets (Yan et al., 2012). Although ^3^H-TCA uptake assay has been developed to measure NTCP uptake activity, ^3^H-TCA is hazardous and very expensive, which significantly limit their usage. Thus, it is of great interest to develop simple and inexpensive uptake assays for NTCP. Because the expression level of NTCP mRNA is low in human hepatocarcinoma cell lines, we employed HepG2 cells stably expressing human NTCP (HepG2 NTCP cells) to evaluate the uptake activity of NTCP (Figure 3A). Using optimized LC-MS/MS and uptake conditions, we performed TCA-d_4_ uptake assay on both HepG2 wildtype and HepG2 NTCP cells. We found that HepG2 NTCP cells was capable of uptaking TCA-d_4_, while wild type cells did not (Figure 3B). We next determined the inhibitory activity of NTCP inhibitor myrcludex B (Gripon et al., 2005) in HepG2 NTCP cells using both TCA-d_4_ and ^3^H-TCA uptake assays. Interestingly, both assays generated similar results with IC_50_ values of 24.2 ± 0.3 nM and 30.7 ± 0.1 nM, respectively, suggesting that our assay was also able to measure NTCP uptake activity (Figures 3C,D).

**FIGURE 4:**
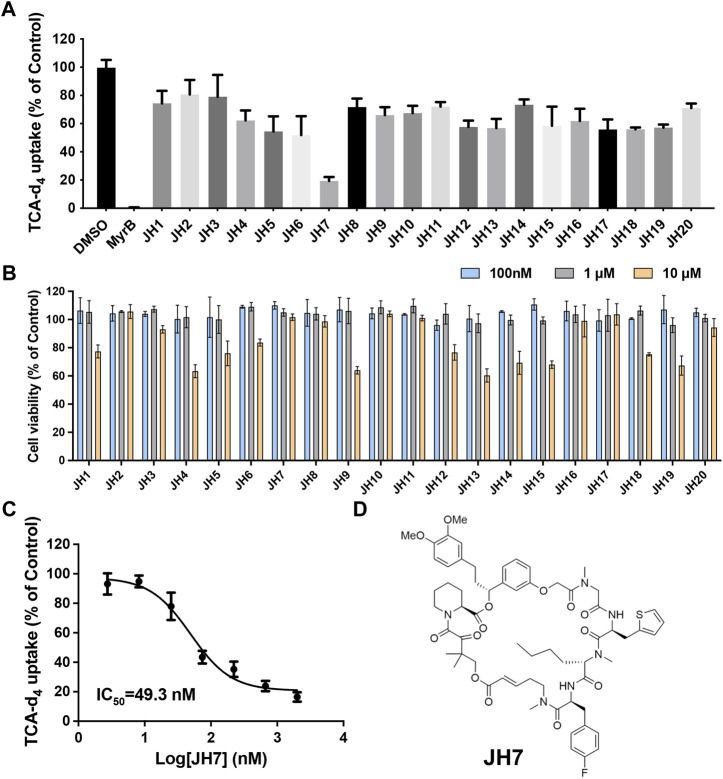
Identification of JH7 as a potential NTCP inhibitor. **(A)** Screening of rapafucins against NTCP using TCA-d_4_ uptake assay in HepG2 NTCP cells. Rapafucins were screened at a final concentration of 2 μM. **(B)** Rapafucins did not show strong inhibition of cell viability in HepG2 NTCP cells. HepG2 NTCP cells were treated with 100 nM, 1 μM and 10 μM of each rapafucin and incubated for 72 h before cell viability was determined. **(C)** Dose-dependent inhibition of NTCP transport activity by JH7 in HepG2 NTCP cells. Error bars represent s.d. Data are presented as mean ± s.d; *n* = 3 independent experiments. **(D)** Structure of JH7.

**FIGURE 5:**
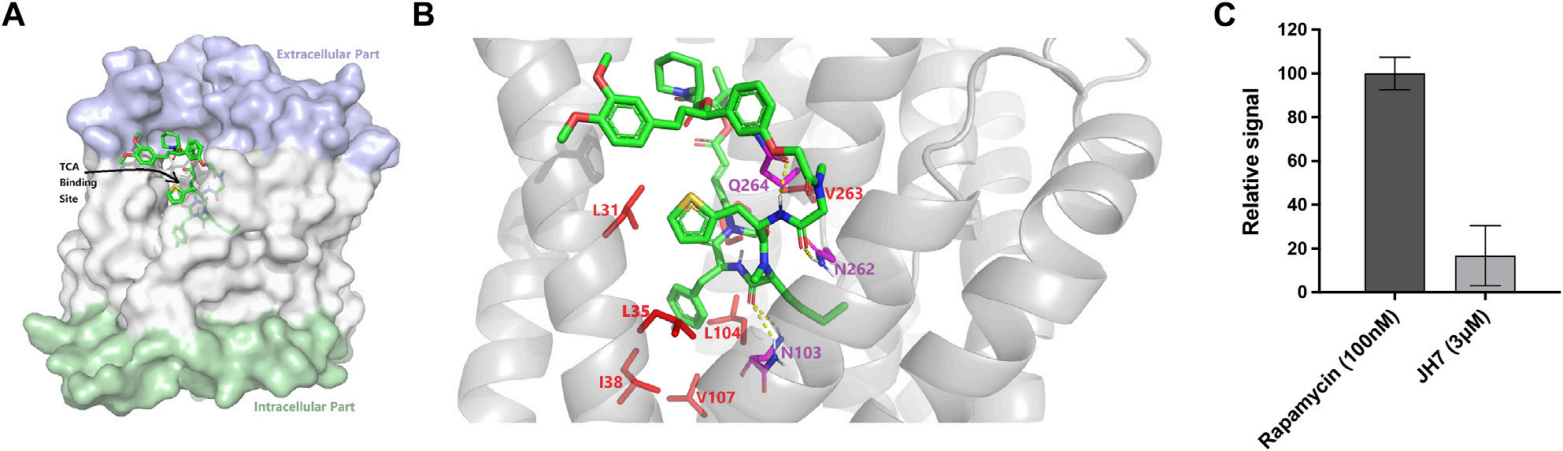
Mechanistic investigations of the inhibition of NTCP by JH7. **(A)** Docked pose of JH7 in predicted binding pocket on NTCP. **(B)** Binding site view of NTCP-JH7. Hydrophobic and polar residues are shown as red and magenta sticks, respectively. Hydrogen bonds are shown as dotted lines. **(C)** Unlike rapamycin, JH7 did not activate BMP reporter gene in HEK 293T cells. Error bars represent s.d. Data are presented as mean ± s.d; *n* = 3 independent experiments.

To further explore the ability of this assay for characterization of modulators, we screened a number of rapafucins for inhibitors of NTCP using TCA-d_4_ uptake assay. We have previously developed a novel class of hybrid macrocycles called rapafucins that were inspired by the unique structure and mode of action of the natural products-derived drugs rapamycin and FK506 (Guo et al., 2019a; Guo et al., 2019b; Park et al., 2022). We borrowed the FKBP-binding domain of rapamycin and replaced its mTOR-interacting domain with a new combinatorial oligopeptide library. Through high-throughput screenings, we have identified two novel inhibitors, named rapadocin and RgA, against equilibrative nucleoside transporter 1 (ENT1, encoded by SLC29A1) and glucose transporter 1 (GLUT1, encoded by SLC2A1), respectively (Guo et al., 2019a; Guo et al., 2019b). To identify potential inhibitors of NTCP, we screened twenty in-house rapafucins using both TCA-d_4_ uptake assay and cell viability assay on HepG2 NTCP cells (Figures 4A,B; Table 2). We found most of rapafucins at a final concentration of 10 μM had slight effects on the cell viability of HepG2 NTCP cells. Interestingly, we identified one hit JH7 from the screen which was able to dose-dependently inhibited TCA-d_4_ uptake with an IC_50_ value of 49.3 ± 0.8 nM, suggesting that JH7 could be a potential novel inhibitor of NTCP (Figures 4C,D). To investigate the mode of action for the JH7-NTCP interaction, we performed binding site prediction for NTCP followed by molecular docking of JH7 (Figures 5A,B). The binding mode showed that JH7 nearly occupied the gateway to the outward-facing tunnel of NTCP. The fluorophenylalanine and neoleucine sidechains within the JH7 effector domain apparently extended to the center of NTCP tunnel where TCA was supposed to bind, resulting in inhibition of TCA uptake. We next employed a BMP reporter gene assay that is dependent on cell penetration of FKBP12 ligands to bind intracellular FKBP12, relieving its inhibition of BMP receptor thus activating the BMP reporter gene (Spiekerkoetter et al., 2013). In comparison to rapamycin, JH7 did not activate the BMP reporter genes, suggesting that JH7 is not capable of penetrating plasma membrane and can only engage NTCP from the extracellular side (Figure 5C). These results demonstrated that JH7 targets the bile acid-binding pocket of NTCP, suggesting a competitive manner of inhibition. To the best of our knowledge, JH7 is one of the most potent small molecule inhibitors of NTCP to date (Chen et al., 2022). This high potency of rapavir is attributable to the macrocyclic scaffold of rapavir and the more extensive contacts it is capable of making with NTCP than conventional small molecule inhibitors of NTCP. It will be interesting to evaluate the anti-HBV activity of JH7 in the future. Together, these results demonstrated that our newly developed assay could be a new tool to screen and characterize modulators of NTCP.

**TABLE 2:** Potency of rapafucins against TCA-d_4_ uptake on HepG2 NTCP cells.

Rapafucin	Amino acid sequences				TCA-d_4_ uptake (% of control)
	Residue 1	Residue 2	Residue 3	Residue 4	
JH1	Phe(4-CF_3_)	mNle	Phe	mGly	74.34
JH2	Phe(4-*t*Bu)	mNle	Phe	mGly	80.64
JH3	PhF	Hyp(tBu)	Phe	mGly	79.07
JH4	PhF	mNle	His	mGly	62.27
JH5	PhF	mNle	dTyr(O-Me)	mGly	54.45
JH6	PhF	mNle	Phe	HoSerMe	51.82
JH7	PhF	mNle	Thien	mGly	19.35
JH8	PhF	mNle	Phe	mIle	71.73
JH9	PhF	mNle	Phe	mIle	66.03
JH10	PhF	mNle	Phe	mSerBu	67.47
JH11	PhF	mNle	Phe	dPhF	72.03
JH12	PhF	mNle	Phe	dPhF	57.67
JH13	PhF	dmPhe	Phe	mGly	56.86
JH14	Phe(4-Cl)	mNle	Tyr	mGly	73.38
JH15	Phe	mNle	Tyr	mGly	58.43
JH16	PhF	Hyp(tBu)	Tyr	mGly	61.79
JH17	PhF	mAla	Tyr	mGly	55.88
JH18	PhF	mNle	Tyr	dmVal	55.96
JH19	PhF	mNle	Tyr	mVal	57.11
JH20	PhF	mNle	Tyr	dmAla	71.05

## 4 Conclusion

In summary, we developed a stable isotope labeled substrate-based LC-MS/MS assay for measuring uptake by SLCs that serves as an alternative to the currently available radiometric or fluorescence-based methods. This novel assay was successfully applied to measure uptake by two SLCs LAT1 and NTCP. Employing stable isotope labeled substrate, this assay circumvents hazardous and costly radioactive materials as well as the limitation of fluorescence substrates and biosensors, and thus greatly improving the usage of SLC uptake assays. Our method is not restricted to LAT1 and NTCP, but can also be used to measure uptake by other SLCs with available stable isotope labeled substrates that can be separated and analyzed by LC-MS/MS. The assay should thus be a valuable tool for investigation of SLCs functional role and characterization of lead compounds that may serve as potential drug candidates for SLC related diseases.

## Data Availability

The original contributions presented in the study are included in the article/Supplementary material, further inquiries can be directed to the corresponding authors.
